# MicroRNA regulate immune pathways in T-cells in multiple sclerosis (MS)

**DOI:** 10.1186/1471-2172-14-32

**Published:** 2013-07-29

**Authors:** Margareta Jernås, Clas Malmeström, Markus Axelsson, Intawat Nookaew, Hans Wadenvik, Jan Lycke, Bob Olsson

**Affiliations:** 1 Department of Molecular and Clinical Medicine, The Sahlgrenska Academy at University of Gothenburg, Gothenburg, Sweden; 2 Department of Neurology, The Sahlgrenska Academy at University of Gothenburg, Gothenburg, Sweden; 3 Department of Internal Medicine, Institute of Medicine, The Sahlgrenska Academy at University of Gothenburg, Vita Stråket 12, SE-413 45, Gothenburg, Sweden; 4 Department of Psychiatry and Neurochemistry, The Sahlgrenska Academy at University of Gothenburg, Gothenburg, Sweden; 5 Department of Chemical and Biological Engineering, Chalmers University of Technology, Gothenburg, Sweden

**Keywords:** Autoimmunity, T-cell, Microarray, MicroRNA

## Abstract

**Background:**

MicroRNA are small noncoding RNA molecules that are involved in the control of gene expression. To investigate the role of microRNA in multiple sclerosis (MS), we performed genome-wide expression analyses of mRNA and microRNA in T-cells from MS patients and controls.

**Methods:**

Heparin-anticoagulated peripheral blood was collected from MS-patients and healthy controls followed by isolation of T-cells. MicroRNA and RNA from T-cells was prepared and hybridized to Affymetrix miR 2.0 array and Affymetrix U133Plus 2.0 Human Genome array (Santa Clara, CA), respectively. Verifications were performed with real-time polymerase chain reaction (PCR) and enzyme-linked immunosorbent assay (ELISA).

**Results:**

We identified 2,452 differentially expressed genes and 21 differentially expressed microRNA between MS patients and controls. By Kolmogorov-Smirnov test, 20 of 21 differentially expressed microRNA were shown to affect the expression of their target genes, many of which were involved in the immune system. Tumor necrosis factor ligand superfamily member 14 (*TNFSF14*) was a microRNA target gene significantly decreased in MS. The differential expression of *mir-494*, *mir-197* and the predicted microRNA target gene *TNFSF14* was verified by real-time PCR and ELISA.

**Conclusion:**

These findings indicate that microRNA may be important regulatory molecules in T-cells in MS.

## Background

Multiple sclerosis (MS) is a chronic inflammatory autoimmune disease characterized by demyelination, astrogliosis and damage to oligodendrocytes and axons in the CNS. The underlying cause and etiology of MS is incompletely understood, but some evidence points to a multifactorial origin involving both central and peripheral immunological tolerance mechanisms. An inappropriate T-cell response to myelin antigens is probably a fundamental mechanism in the MS pathophysiology [1,2].

MicroRNA are 19–25 base pair long single stranded RNA nucleotides. They are evolutionary conserved and are increasingly regarded as being involved in all aspects of cellular function in a wide range of eukaryotic organisms [3]. Approximately 33% of the human genes are estimated to be regulated by microRNA and a single microRNA can potentially regulate multiple mRNA targets (in average 200) [4,5]. There are more than 1500 human microRNA identified (http://www.mirbase.org) [6] and they are associated with major pathologies such as autoimmune diseases [7-9] and cancer [10]. However, the *in vivo* function of most microRNA is largely unknown.

MicroRNA are highly expressed in cells of the immune system [11,12] and they are involved in numerous pathways of both the innate and the adaptive immune system [3,13-16]. It has been reported that microRNA are critical for the maintenance of immunological tolerance; deletion of the enzymes Dicer- and Drosha that are involved in the generation of microRNAs, results in T-cell abnormalities and autoimmune diseases [17,18]. The microRNA transcriptome of immune cell subsets are distinct, suggesting that naïve, effector and central memory T cell [19] and regulatory T cell function [17,18,20] are dependent on microRNA regulation. Furthermore, microRNA are reported to be important regulatory factors in the differentiation of T cells [21] and associated with activation of T cell-mediated immune responses [22]. Several publications have addressed the role of individual microRNAs in MS [23-34]. However, very few have performed large scale unbiased studies. Furthermore, most studies have investigated microRNA expression in peripheral blood mononuclear cells (PBMC). PBMC is a compartment with heterogeneous cell composition due to individual biological variation or disease state. Therefore, the aim of this study was to identify differentially expressed microRNA and target mRNA in T-cells from MS-patients using global DNA microarray analyses.

## Methods

### Participants

All individuals involved in this study gave informed consent. The study was approved by the regional ethics committee in Gothenburg, Sweden.

The ten relapsing remitting MS (RRMS) subjects in the mRNA analysis (six males and four females; age 38.1 ± 11.7 years) have previously been described [35]. For the microRNA experiment, heparin-anticoagulated peripheral blood was obtained from 11 (1 male, 10 females, mean age 42.9 ± 3.4 years) relapsing-remitting MS (RRMS) patients according to the McDonald criteria [36,37] who were on treatment with beta interferon (Additional file 1: Table S1). The disease duration was 10.6 ± 2.1 years and neurological deficits were scored with Expanded Disability Status Scale (EDSS) [38] and median was 2.1 (range 0–6). Nine healthy individuals served as controls (4 males, 5 females mean age 42.6 ± 13.6).

For the verification of microRNA experiments by real-time PCR heparin-anticoagulated peripheral blood was collected from 16 RRMS patients (1 male, 15 females, mean age 45.1 ± 2.7) according to the McDonald criteria [36,37]. The disease duration was 12.0 ± 1.9 years and the median EDSS score was 2.4 (range 0–6). Twelve healthy individuals served as controls (6 males, 6 females, mean age 44.4 ± 12.0). For the verification of mRNA expression of *TNFSF14* by real-time PCR analysis, the same patients were used, however there were only RNA left from 13 patients (Additional file 1: Table S1).

For the ELISA experiment, peripheral blood without anticoagulantia was collected from 16 RRMS patients (1 male, 15 females, mean age 45.1 ± 2.7) according to the McDonald criteria [36,37]. The disease duration was 12.0 ± 1.9 years and the median EDSS score was 2.4 (range 0–6) (Additional file 1: Table S1). Twenty healthy individuals served as controls (6 males, 14 females, mean age 38.4 ± 12.0).

### Isolation of T-cells and preparation of RNA

The isolation of T-cells has previously been described [39]. In brief, peripheral blood mononuclear cells (PBMCs) were separated from heparinized blood immediately after collection, by density gradient centrifugation using Ficoll-Paque PLUS (GE Healthcare, Uppsala, Sweden). After removal of CD14^+^ cells by magnetic microbeads, T-cells were positively selected using CD3^+^ magnetic microbeads, according to the manufacturer's recommendations (MACS, Miltenyi Biotec, Surrey, UK). The purity of isolated cells was thereafter determined by flow cytometry; 93.6-96.2% of the cells were CD3+ T-cells, with no significant differences between the study groups. The cells were frozen and stored at −80°C. RNA was isolated from the CD3^+^ T-cells using the Chomczynski method [40]. For the microRNA analysis the RNA was precipitated using isopropanol overnight. For the DNA microarray analysis the RNA was further purified using RNeasy MinElute clean-up (Qiagen, Hilden, Germany). The RNA concentration was measured with a Nanodrop spectrophotometer and the A260/A280 ratio was 1.8–2.0. The quality of the RNA was verified by agarose gel electrophoresis for the DNA microarray analysis. The mean quantity of total RNA from T-cells in peripheral blood was 5.8 μg (range 1.2-10.3 μg) for the microRNA analysis and for the DNA microarray analysis 36.8 μg (1.08-104.88 μg).

### Labeling and hybridization to microRNA 2.0 microarrays

1000 ng RNA from T-cells were biotin labeled using the FlashTag Biotin HSR kit (Genisphere, Hatfield, PA) according to the manufacturer's instructions. The labeled microRNA was controlled using the Enzyme Linked Oligosorbent Assay (ELOSA) step (Genisphere), following the manufacturer’s instructions. All samples showed expected labeling and the resulting targets were hybridized to miR 2.0 arrays (Affymetrix), containing 15,644 microRNA probe sets from the miRBASE v15 (http://microrna.sanger.ac.uk). The arrays recognize microRNA from 131 organisms, including human small nucleolar RNA such as snoRNA and small Cajal body-specific RNA (scaRNA) from the Ensembl Archive (http://www.ensembl.org/biomart/martview) and snoRNAbase (http://www-snorna.biotoul.fr/info.php). The arrays were washed and processed on a Fluidics Station 450 and scanned with a confocal laser scanner (GeneChip Scanner 3000, Affymetrix) according to the manufacturer's instructions.

### Labeling and hybridization to U133Plus 2.0 DNA microarrays

As previously described 5.0 ug RNA was reverse transcribed into cDNA (Superscript II, Invitrogen, Carlsbad, CA) [35]. Biotin-labeled target cRNA was prepared from cDNA by in vitro transcription (Enzo Diagnostics, Farmingdale, NY) and hybridized to Human Genome HG-U133 plus 2.0 arrays (Affymetrix, Santa Clara, CA), containing 54,675 probe sets, as recommended in the Affymetrix Gene Chip Expression Analysis manual. Arrays were scanned with a confocal laser scanner (GeneChip Scanner 3000, Affymetrix) and visualized using GCOS. These experiments comply with Minimum Information About a Microarray Experiment (MIAME) [41].

### Data analysis of miR_2.0 microarrays

Data from the microarrays were analyzed with Affymetrix microRNA QC Tool according to the manufacturers’ instructions. Robust Multi-array Analysis (RMA) was used for normalization. To be included in the analysis the probe sets had to be detected in more than 50% of the patients and controls. Thereafter, the differential gene expression was evaluated using Student’s t-test (two tailed).

### Data analysis of DNA microarrays U133Plus 2.0

**Figure 1:**
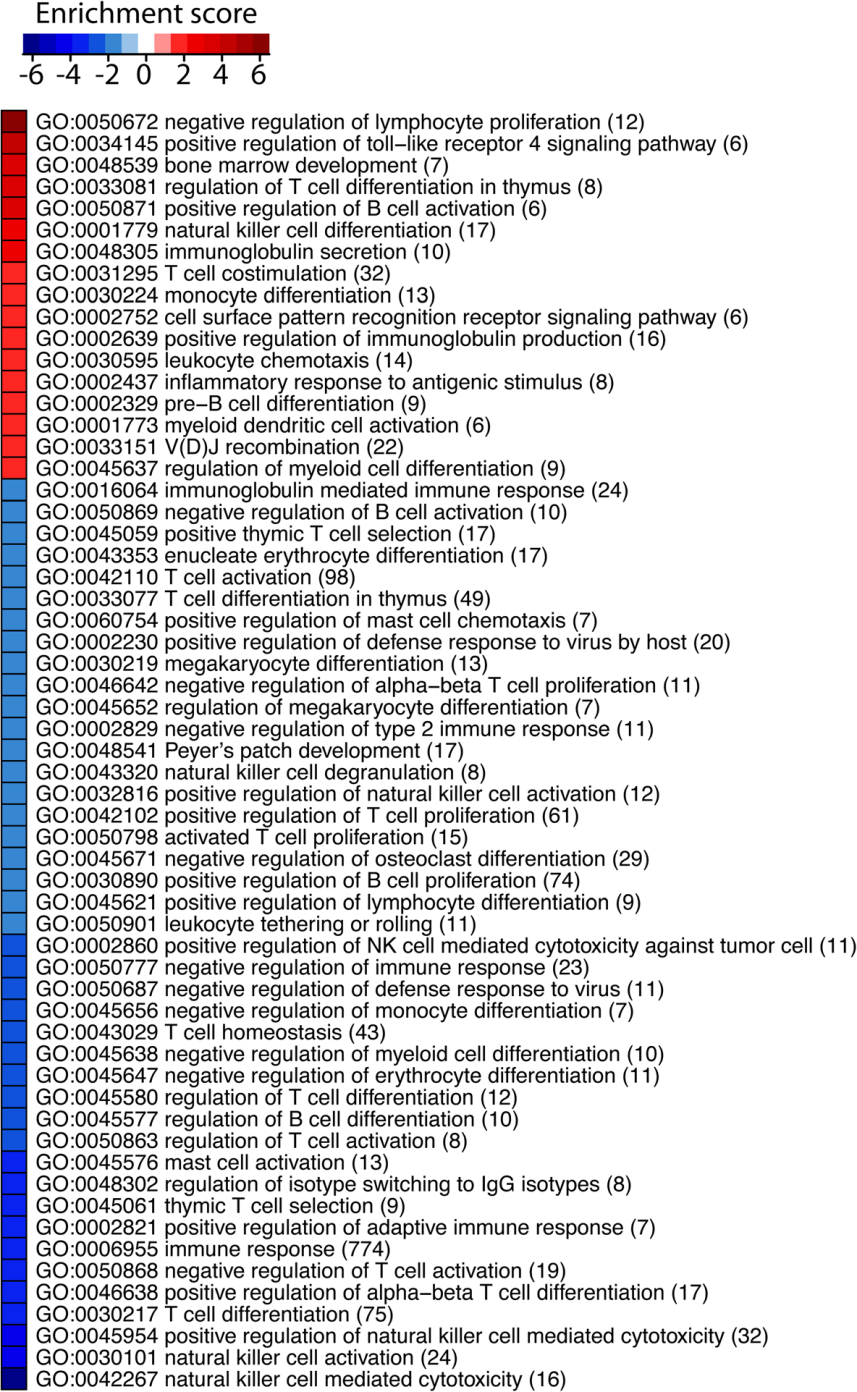
**In the DNA microarray analysis we identified 2452 differentially expressed genes (*Q*** **< 0.05; PLIER) in peripheral blood T-cells that differed between MS patients and healthy controls.** In order to identify which biological processes the differentially expressed genes belonged to we used GO. Two hundred and fifty-six genes were classified as immune-related according to GO [53]. The number of genes in each category is shown in parenthesis. The enrichment score (−log10 (enrichment *P*-values)) is shown in red for overrepresented gene ontology terms and in blue for the ones underrepresented in MS patients compared with controls. All gene ontology terms that had *P*-values < 0.05 are shown.

The DNA and microRNA microarrays (GSE43592) were processed and normalized together using Probe Logarithmic Intensity Error (PLIER) method [42]. Differences in gene expression between the groups were investigated by Student’s *t*-test (two tailed) using linear models together with empirical Bayes [43]. P-values from the student’s *t*-test were transformed into Q-values through the correction for multiple testing [44]. The Q-values were overlaid on Gene Ontology (GO) networks and then a reporter algorithm [45,46] was used to evaluate the enrichment p-value of each GO term. GO terms that had enrichment P-values < 0.05 were selected in the construction of a heatmap as illustrated in Figure 1. The analyses were performed using piano package [47] under the R software environment.

### Computational methods to identify microRNA functions and mRNA targets

The most commonly used microRNA database mirBase (http://www.mirbase.org) released in November 2011 [6], including over 21643 mature microRNA products in 168 species, was used to identify microRNA functions and target mRNAs. Multiple computational methods are developed to predict microRNA target sites [48] and in general, target prediction algorithms focus on the complementarities between the microRNA and the potential targets around the “seed” sequence, heteroduplex free energy of binding, location and size of internal loops and bulges, and accessibility of the target site as predicted by RNA folding. To predict mRNA targets to the differentially expressed microRNAs in the present study, we used TargetScan [49] (http://www.targetscan.org) and Miranda [50] (http://www.microrna.org/microrna/getDownloads.do). However, there are some evidence that perfect seed pairing may not necessarily be a reliable prediction for microRNA-mRNA interaction since these bioinformatics tools do not take into account the secondary structure of the mRNA which may affect the microRNA target recognition [51]. Therefore, as a way to achieve the high confidence microRNA-mRNA associations and evaluate the impact of each microRNA on the gene expression, the predicted microRNA target genes of each microRNA were identified and combined with mRNA transcriptome from MS patients and controls. Kolmogorov-Smirnov test was employed to evaluate the impact of each microRNA on gene expression between MS patients and controls (the test was performed over cumulative fraction of gene expression fold changes of specific microRNA targets and background, which is the rest of the genes that do not contain the targets of specific microRNA [5,49]). The results are reported in Table 1. The identified target genes were cross referenced against the differentially expressed mRNAs that differed between MS patients and controls.

**Table 1:** Differentially expressed small nucleolar RNA in T-cells between MS patients and controls

**Small nucleolar RNA**	**Fold change MS/Control**	**P-value**
mgU12-22-U4-8_s_st	0.66	0.037
mgU2-25-61_s_st	0.84	0.041
SNORD119_st	0.77	0.03
U104_st	0.77	0.027
U105_st	0.83	0.046
U105B_st	0.39	0.023
U17b_st	0.53	0.025
U17b_x_st	0.58	0.02
U27_st	0.57	0.004
U27_x_st	0.55	0.01
U31_st	0.71	0.031
U31_x_st	0.69	0.035
U42B_x_st	0.79	0.042
U56_st	0.43	0.031
U56_x_st	0.33	0.005
U58A_st	0.85	0.02
U59A_st	0.76	0.014
U61_st	0.66	0.01
U79_st	0.81	0.028
U80_st	0.70	0.018
U83_st	0.65	0.02
U83A_x_st	0.80	0.03
U84_x_st	0.75	0.029
v11_hsa-miR-768-3p_st	0.74	0.028
v11_hsa-miR-768-5p_st	0.86	0.025
ACA16_st	0.76	0.014
ACA16_x_st	0.69	0.002
ACA24_x_st	0.68	0.008
ACA44_s_st	0.50	0.048
ACA48_x_st	0.68	0.005
ACA54_st	0.45	0.003
ACA7_s_st	0.40	0.002
ACA7B_s_st	0.52	0.01
E3_x_st	0.73	0.016
ENSG00000201009_s_st	0.58	0.033
ENSG00000206903_s_st	0.54	0.031
ENSG00000206913_s_st	0.40	0.002
ENSG00000212615_x_st	0.77	0.014
ENSG00000239031_st	1.15	0.039
ENSG00000252049_st	1.10	0.046
HBII-210_st	0.42	0.028
HBII-436_st	0.81	0.018
HBII-55_st	0.62	0.04
HBII-85-17_x_st	0.85	0.031
HBII-85-29_st	0.65	0.004
HBII-85-3_x_st	0.81	0.003
HBII-85-9_x_st	0.87	0.043
HBII-99_st	0.51	0.011
hp_hsa-let-7d_st	0.90	0.044
hp_hsa-mir-423_s_st	0.52	0.023

Displayed is the fold change in expression between MS patients and controls.

### Real-time PCR analysis of microRNA expression

*Mir-494* and *mir-197* were selected for verification and specific Taqman® MicroRNA assays (Applied Biosystems, Life Technology, Foster City, CA) were used. Reverse transcriptase (RT) reactions contained 10 ng RNA, 5 X stem–loop RT primer, 10× RT buffer, 100 mM dNTPs, 50 U/μl MultiScribe reverse transcriptase and 20 U/μl RNase inhibitor (Applied Biosystems). The reactions were incubated for 30 min at 16°C, 30 min at 42°C, 5 min at 85°C. The High Capacity RNA-to-cDNA Kit (Applied Biosystems) was used for the housekeeping-gene *RPLP0*. All reverse transcriptase reactions were run in duplicate.

Real-time PCR was performed using a standard TaqMan® PCR kit protocol on an Applied Biosystems 7900HT Sequence Detection System. The PCR included the RT product, 1× TaqMan® Universal PCR Master Mix and TaqMan® Small RNA assay (20 ×). The reactions were incubated at 95°C for 10 min, followed by 40 cycles of 95°C for 15 s and 60°C for 1 min. The cycle threshold (Ct) value of the house-keeping gene *RPLP0* was subtracted from the corresponding Ct value for mir-494 and mir-197 resulting in the ΔCt value and relative quantification was calculated using the (2^-ΔΔCT^) formula [52]. Differences were analyzed by Student’s *t*-test (two tailed).

### Real-time PCR analysis of mRNA expression of *TNFSF14*

Reagents for real-time PCR analysis of *TNFSF14*, such as High Capacity RNA-to-cDNA Kit and TaqMan Universal PCR Master Mix, were obtained from Applied Biosystems by Life Technologies (Carlsbad, CA) and used according to the manufacturer’s protocol. *RPLP0* was used as reference to normalize the expression levels between samples. cDNA was synthesized from 200 ng of total RNA in a reaction volume of 20 μl. Specific products were amplified and detected with the ABI Prism 7900HT Sequence Detection System (Applied Biosystems) using default cycle parameters. All samples were analysed in triplicates. The relative comparative *C*_T_ method was used to analyse the real-time PCR data [52]. Differences were analyzed by Students T-test.

### Immunoassay analysis of TNFSF14

Serum levels of TNFSF14 were analyzed with a commercial ELISA from R&D systems (Minneapolis, MN). Intra-assay coefficient of variation was 5.1% and the limit of detection was 31 ng/l. Differences were analyzed by Students T-test.

### Statistics

The statistics for each analysis is given above in their subsections. *P*-values ≤ 0.05 were considered significant and all data are presented as mean ± SEM unless otherwise stated.

## Results

### Differentially expressed microRNA in T-cells between MS patients and controls

To identify T-cell microRNA with a differential expression between MS patients and controls we analyzed approximately 1000 microRNA with microarray analysis. On the microarrays were also probes for a large number of small snoRNA and scaRNA. Twenty-one microRNA had decreased expression in peripheral blood T-cells in MS patients compared with controls (Table 2). The mature microRNA sequences are given in (Additional file 1: Table S2). In addition, 50 small nucleolar RNA such as snoRNA and scaRNAs were differently expressed in T-cells between MS patients and controls (Table 1).

**Table 2:** Impact of differentially expressed microRNA on gene expression in T-cells between MS patients and controls

**microRNA**	**microRNA microarray**		**Impact of microRNA on its targets (mRNA microarray)**	
	**Fold change MS/Control**	***P-value***	**TargetScan**	**Miranda**
hsa-miR-494	0.14	3.1 × 10^-05^	1.2 × 10^-24^	6.0 × 10^-81^
hsa-miR-15b	0.14	2.5 × 10^-03^	4.1 × 10^-34^	5.5 × 10^-15^
hsa-miR-30c	0.15	7.7 × 10^-04^	2.8 × 10^-13^	5.0 × 10^-58^
hsa-miR-23a	0.16	7.8 × 10^-03^	2.2 × 10^-23^	9.8 × 10^-63^
hsa-miR-197	0.19	5.2 × 10^-06^	1.6 × 10^-12^	1.2 × 10^-06^
hsa-miR-1260b	0.21	9.4 × 10^-07^	NA	2.3 × 10^-16^
hsa-miR-125a-5p	0.22	8.2 × 10^-03^	1.2 × 10^-43^	5.3 × 10^-13^
hsa-miR-361-5p	0.24	1.4 × 10^-02^	1.8 × 10^-15^	3.9 × 10^-32^
hsa-miR-320d	0.26	2.8 × 10^-03^	2.0 × 10^-09^	7.4 × 10^-36^
hsa-miR-423-3p	0.26	1.6 × 10^-02^	5.0 × 10^-13^	1.8 × 10^-03^
hsa-miR-1280	0.29	4.7 × 10^-03^	8.2 × 10^-56^	8.6 × 10^-04^
hsa-miR-663	0.32	3.5 × 10^-03^	5.9 × 10^-70^	1.8 × 10^-28^
hsa-miR-423-5p	0.33	2.6 × 10^-02^	6.1 × 10^-96^	5.7 × 10^-36^
hsa-miR-99b	0.36	2.4 × 10^-02^	3.4 × 10^-04^	7.6 × 10^-03^
hsa-miR-339-5p	0.37	4.9 × 10^-02^	1.5 × 10^-35^	2.5 × 10^-07^
hsa-let-7a	0.37	2.9 × 10^-02^	1.9 × 10^-04^	3.2 × 10^-09^
hsa-miR-1979	0.38	1.2 × 10^-02^	NA	8.1 × 10^-09^
hsa-miR-3178	0.39	1.0 × 10^-02^	NA	NS
hsa-miR-625	0.41	3.2 × 10^-02^	3.6 × 10^-62^	2.3 × 10^-09^
hsa-miR-150	0.65	3.7 × 10^-02^	2.1 × 10^-68^	2.1 × 10^-11^
hsa-miR-3153	1.14	4.5 × 10^-02^	NA	2.4 × 10^-09^

To investigate the impact of the significantly changed microRNAs on gene expression in T-cells we performed Kolmogorov-Smirnov test using the target genes identified in TargetScan and Miranda. NS: not significant, NA: not available in the database. Displayed is the fold change in expression between MS patients and controls.

### Differentially expressed mRNA in T-cells between MS patients and controls

DNA microarray analysis was used to identify differentially expressed T-cell genes between MS patients and controls. In the DNA microarray analysis we identified 2452 differentially expressed genes (*Q* < 0.05; PLIER) in peripheral blood T-cells that differed between MS patients and healthy controls. In order to identify which biological processes the differentially expressed genes belonged to we used GO. Two hundred and fifty-six genes were classified as immune-related according to GO [53] and over or under represented biological processes for these genes are shown in Figure 1.

### Identification of differentially expressed target genes of microRNA

To better understand the role of the differentially expressed microRNA in MS patients we first identified the predicted target genes for each of the microRNA using the databases TargetScan and Miranda. Second, to investigate if the significantly changed microRNA had any effect on gene expression we performed Kolmogorov-Smirnov test using the target genes identified in TargetScan and Miranda on the gene expression data. The results showed that there were some differences between the two databases TargetScan and Miranda but that in most cases the same results were obtained, i.e. that the differently expressed microRNA did indeed significantly affect the gene expression (Table 2). Third, to identify which of the differentially expressed genes that were indeed target genes of the differentially expressed microRNA we compared the list of microRNA target genes with our list of differentially expressed genes between MS and controls. This resulted in 920 genes. Fourth, these 920 genes were classified according GO and 100 genes were classified as being involved in immune processes. The top 25 genes with increased and decreased expression between MS and controls, respectively, are shown in Additional file 1: Table S3. They were further analyzed using functional module enrichment [54] based on Immune System Gene Ontology [53] which resulted in 21 modules that were enriched with separate functions (see following list and Additional file 1: Figure S1).

List of functional module enrichment based on Immune System Gene Ontology on target genes of differentially expressed microRNA between MS patients and controls:

Antigen processing and presentation of exogenous peptide antigen via MHC class I, TAP-dependent

T cell migration

Megakaryocyte differentiation

Toll-like receptor 3 signaling pathway

Regulation of response to interferon-gamma

Natural killer cell mediated immunity

Negative regulation of lymphocyte activation

Negative regulation of inflammatory response to antigenic stimulus

Regulation of inflammatory response to antigenic stimulus

Thymic T cell selection

Natural killer cell differentiation

V(D)J recombination

Megakaryocyte differentiation

Negative regulation of erythrocyte differentiation

Megakaryocyte differentiation

Negative regulation of type 2 immune response

Dendritic cell chemotaxis

Natural killer cell activation

Regulation of defense response to virus

Positive regulation of monocyte chemotaxis

Regulation of toll-like receptor 3 signaling pathway

**Figure 2:**
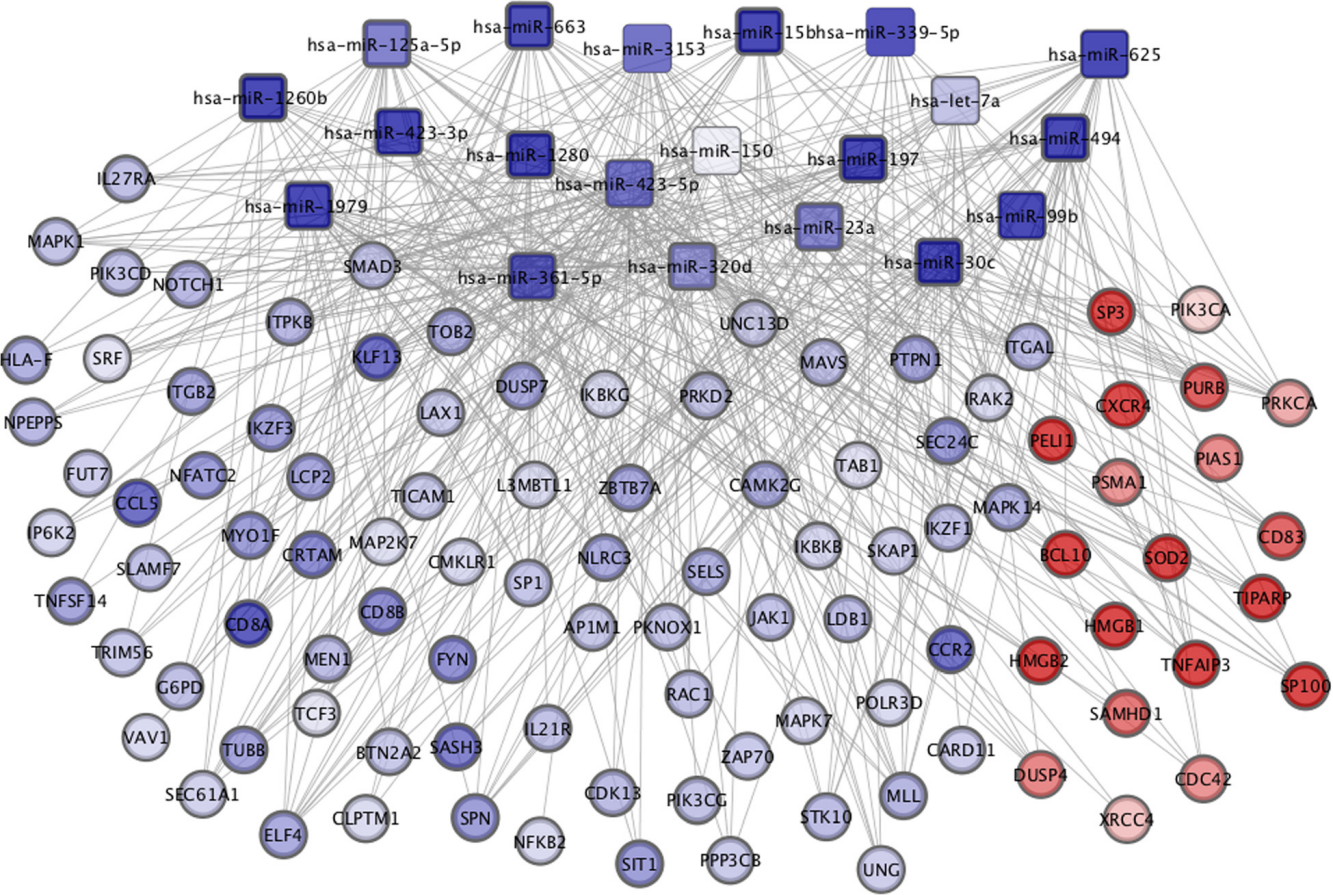
**A computational method to identify microRNA function and mRNA targets in T-cells.** Target genes of significantly regulated microRNA between MS patients and controls, using TargetScan and Miranda algorithms, were compared with significantly differently expressed mRNA from peripheral blood T-cells between patients and controls. Squares indicate microRNA and circles mRNA. Red indicates increased expression and blue decreased expression between MS patients and controls.

The interactions between the 20 microRNA and the 100 genes are shown in Figure 2.

### Verification of a differently expressed microRNA using real-time PCR

**Figure 3:**
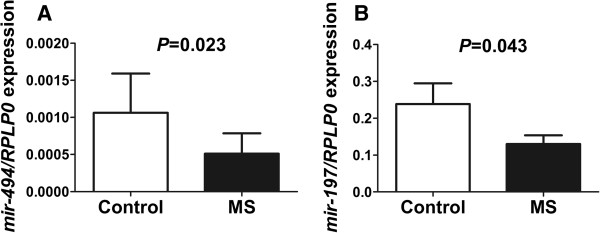
**Verification of the differential expression of A) *mir-494 *and B) *mir-197 *between MS patients (n = 16) and healthy controls (n = 12) by real-time PCR.** Data are presented as mean ± SEM.

To verify the results from the DNA microarray analysis we selected *mir-494* and *mir-197* because they were among the microRNAs that had the largest fold change in expression between MS patients and controls of all the microRNA (Table 1). We analyzed the expression of *mir-494* and *mir-197* with real-time PCR and confirmed that the expression was significantly decreased in MS patients compared with controls (*P* = 0.02 and *P* = 0.04; Figure 3A-B).

### Verification of *TNFSF14* using real-time PCR

**Figure 4:**
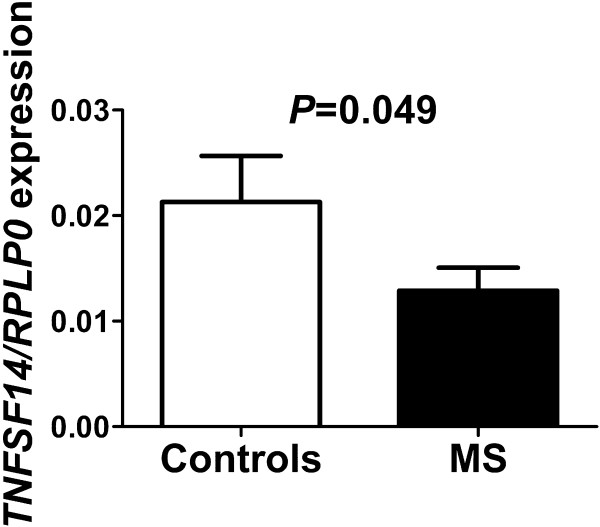
**Verification of the differential mRNA expression of *TNFSF14 *between the MS patients (n = 13) and healthy controls (n = 9).** Data are presented as mean ± SEM.

To verify the results in the DNA microarray analysis and predicted mRNA target analysis, we analyzed the expression of *TNFSF14* with real-time PCR and confirmed that the expression was significantly decreased in MS patients compared with controls (*P* = 0.05; Figure 4).

### Serum levels of TNFSF14 in MS compared with healthy controls

**Figure 5:**
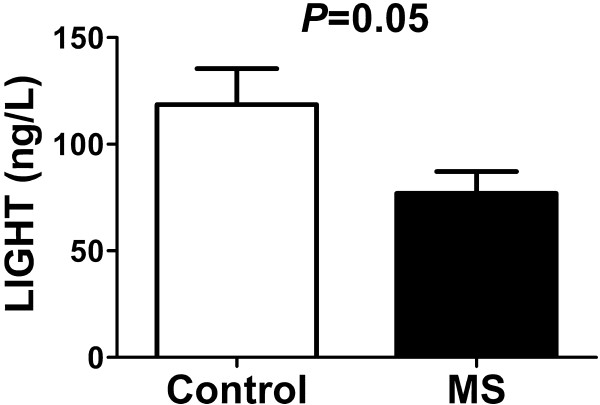
**Verification of the protein levels of TNFSF14 between the MS patients (n = 16) and healthy controls (n = 20).** Data are presented as mean ± SEM.

To test whether changes in microRNA expression were accompanied by changes of corresponding proteins we determined serum levels of TNFSF14 in MS patients and controls. The serum level of this protein was found to be significantly decreased in MS patients compared with controls (*P* = 0.05; Figure 5).

## Discussion

To explore genes and mechanisms involved in MS we performed global microarray analyses of mRNA and microRNA in T-cells from MS patients and controls. We found 2,452 genes and 21 microRNA that differed significantly in expression between MS patients and controls. Of these, 20 microRNA significantly affected the expression of their target genes. When the microRNA target genes were cross-referenced against the genes that differed in expression between MS patients and controls 920 genes were identified and 100 were involved in the immune system. To better understand the role of the target genes we clustered the genes involved in the immune system using GO. The significantly enriched immune processes observed between MS and controls, points to well known processes within autoimmunity. Four of the 21 (mir-150, mir-15b, mir-1979 and mir-23a) identified microRNA in this study have already been linked to MS, however, not in CD3^+^ T-cells [27,28,34,55]. We also found 50 small nucleolar RNAs such as snoRNAs and scaRNAs that were differently expressed in T-cells between MS patients and controls. However, in this manuscript we have focused on microRNA since the amount of information concerning the smaller molecules such as snoRNA is scarce to say the least.

We found several genes that were involved in immunological pathways previously associated with MS such as ITGAM, CD8A, CD8B, CCL5, CCR2, TGFBR1 and TNFSF14 among the top genes regulated by the differentially expressed microRNA. ITGAM is an integrin involved in leukocyte migration that is expressed on T-cells and other leukocytes [56]. ITGAM expression is critical on T-cells and phagocytes for the development of EAE in mice [57]. CD8A and CD8B are the two genes that codes for the subunits of CD8 which is specifically expressed on cytotoxic T-cells which are involved in major histocompatibility class I immunosurveillance. CCL5, also known as RANTES, is a chemokine and T-cells from MS patients have been shown to have increased migration towards CCL5 compared with T-cells from healthy controls [58]. Furthermore, treatment with anti-CCL5 antibodies in EAE mice prevented leukocyte adhesion but not rolling [59] and a selective antagonist against one of the receptors for CCL5, namely CCR1, abrogated clinical disease signs in EAE rats [60]. CCR2 is the receptor for CCL2, also known as monocyte chemoattractant protein 1 (MCP-1). Anti-CCL2 treatment prevented the development of paralysis in EAE mice [61] and leukocyte adhesion [59]. TGFBR1 is the receptor for TGF-β1 and administration of TGF-β1 protects and even improves the clinical course of EAE when given during ongoing disease [62]. Furthermore, deletion of TGF-β1 in mice leads to an early death caused by massive tissue infiltration of lymphocytes and macrophages resembling autoimmunity [63].

*TNFSF14* is a newly identified risk gene for MS along with its receptor *TNFRSF14* (*HVEM*). A recent study showed that membrane-bound TNFSF14 is important for CD4^+^ memory T-cell survival [64]. Membrane-bound TNFSF14 is a co-stimulatory molecule [65] which has diverse stimulatory effects on the immune system. Over-expression of the membrane-bound form leads on T-cells leads to autoimmunity in mice [66-68]. It has also been implicated in asthma and tumor response [69,70]. In contrast, the soluble form of TNFSF14 has been shown to act as an inhibitor of activation [71-74]. We chose TNFSF14 for verification by real-time PCR and ELISA because it had a high expression level and the fact that there was a soluble form of the protein. Indeed, we found that both the mRNA and the protein expression of the microRNA target gene TNFSF14 was decreased in MS compared with controls.

Limitations of this study include the lack of naïve MS patients and the lack of gender matching.

## Conclusion

These findings indicate that microRNA may be important regulatory molecules in T-cells in MS.

### Reviewer link to access microarray data

http://www.ncbi.nlm.nih.gov/geo/query/acc.cgi?token=blapdgogguqwklw&acc=GSE43592.

## Competing interests

MJ, MA, IN, HW and BO do not have any competing interests in this study. CM has received travel grants and lecture fees from BiogenIdec and Merck and compensation for advisory board participation from Novartis. JL has served on advisory boards for Biogen Idec and Merck Serono; has grants pending from Biogen Idec and Novartis; has received speakers honoraria from Biogen Idec, Merck Serono, TEVA and Novartis; and has had travel expenses reimbursed by Biogen Idec.

## Authors’ contribution

MJ designed and coordinated the study, performed all laboratory work, analyzed and interpreted the data and wrote the paper. IN analyzed and interpreted data and wrote the paper. CM and MA collected the patient material, interpreted the data and wrote the paper. HW and JL interpreted the data and wrote the paper. BO designed and coordinated the study, analyzed and interpreted data and wrote the paper. All authors read and approved the final manuscript.

## Supplementary Material

Additional file 1: Table S1 Patient characteristics in the microRNA analysis. **Table S2.** Significantly regulated microRNAs in T-cells between MS patients and controls. **Table S3.** Differentially expressed mRNAs in T-cells between MS patients and controls. **Figure S1.** The identified target genes of the differentially expressed microRNA were cross referenced against the differentially expressed mRNAs between MS patients and controls resulting in 920 overlapping genes. Click here for file
