# Supplementary material for: MicroRNA regulate immune pathways in T-cells in multiple sclerosis (MS)
Source: BMC Immunol. 2013 Jul 29;14:32. doi: 10.1186/1471-2172-14-32 (PMC3734042; doi:10.1186/1471-2172-14-32)
Supplement: Additional file 1: Table S1 — Patient characteristics in the microRNA analysis. Table S2. Significantly regulated microRNAs in T-cells between MS patients and controls. Table S3. Differentially expressed mRNAs in T-cells between MS patients and controls. Figure S1. The identified target genes of the differentially expressed microRNA were cross referenced against the differentially expressed mRNAs between MS patients and controls resulting in 920 overlapping genes. [file 1471-2172-14-32-S1.doc]

**Supplemental Table 1.**

Patient characteristics in the microRNA analysis.

| **Gender** | **Age**  **(years)** | **Duration**  **(month)** | **EDSS** | **Array** | **Real-time PCR microRNA** | **ELISA** | **Real-time PCR mRNA** |
| --- | --- | --- | --- | --- | --- | --- | --- |
| F | 55 | 21 | 6 | X | X | X | X |
| F | 38 | 2 | 1.5 | X | X | X | X |
| F | 28 | 5 | 2.5 | X | X | X | X |
| F | 39 | 4 | 0 | X | X | X | X |
| F | 36 | 9 | 2 | X | X | X | X |
| F | 58 | 15 | 3 | X | X | X | X |
| F | 51 | 1 | 2 | X | X | X | X |
| F | 37 | 16 | 2 | X | X | X | X |
| F | 37 | 13 | 0 | X | X | X | X |
| M | 61 | 18 | 4 | X | X | X | X |
| F | 33 | 13 | 0 | X | X | X | X |
| F | 55 | 14 | 6 |  | X | X |  |
| F | 59 | 25 | 4.5 |  | X | X |  |
| M | 41 | 7 | 0 |  | X | X |  |
| M | 40 | 5 | 3 |  | X | X | X |
| F | 53 | 24 | 2 |  | X | X | X |

RRMS = relapsing remitting multiple sclerosis, EDSS = Expanded Disability Status Scale.

EDSS median (range) is given.

**Supplemental Table 2.**

### Significantly regulated microRNAs in T-cells between MS patients and controls.

Twenty-one microRNA were differentially expressed in peripheral blood T-cells between MS patients and controls. The table shows the mature microRNA sequences.

| **microRNA** | **Mature microRNA sequences** |
| --- | --- |
| hsa-let-7a | UGAGGUAGUAGGUUGUAUAGUU |
| hsa-miR-125a-5p | UCCCUGAGACCCUUUAACCUGUGA |
| hsa-miR-1260b | AUCCCACCACUGCCACCAU |
| hsa-miR-1280 | UCCCACCGCUGCCACCC |
| hsa-miR-150 | UCUCCCAACCCUUGUACCAGUG |
| hsa-miR-15b | UAGCAGCACAUCAUGGUUUACA |
| hsa-miR-197 | UUCACCACCUUCUCCACCCAGC |
| hsa-miR-1979 | CUCCCACUGCUUCACUUGACUA |
| hsa-miR-23a | AUCACAUUGCCAGGGAUUUCC |
| hsa-miR-30c | UGUAAACAUCCUACACUCUCAGC |
| hsa-miR-3153 | GGGGAAAGCGAGUAGGGACAUUU |
| hsa-miR-3178 | GGGGCGCGGCCGGAUCG |
| hsa-miR-320d | AAAAGCUGGGUUGAGAGGA |
| hsa-miR-339-5p | UCCCUGUCCUCCAGGAGCUCACG |
| hsa-miR-361-5p | UUAUCAGAAUCUCCAGGGGUAC |
| hsa-miR-423-3p | AGCUCGGUCUGAGGCCCCUCAGU |
| hsa-miR-423-5p | UGAGGGGCAGAGAGCGAGACUUU |
| hsa-miR-494 | UGAAACAUACACGGGAAACCUC |
| hsa-miR-625 | AGGGGGAAAGUUCUAUAGUCC |
| hsa-miR-663 | AGGCGGGGCGCCGCGGGACCGC |
| hsa-miR-99b | CACCCGUAGAACCGACCUUGCG |

**Supplemental Table 3.** Differentially expressed mRNAs in T-cells between MS patients and controls. The top 25 immune genes that were targets of microRNA with differential expression between MS patients and controls.

| **Gene symbol** | **logFC (MS/Control)** | **P-value** | **Q-value** |
| --- | --- | --- | --- |
| ITGAM | -0.93 | 2.56E-04 | 9.07E-03 |
| CD8A | -0.91 | 2.40E-05 | 1.93E-03 |
| CCL5 | -0.83 | 6.53E-04 | 1.68E-02 |
| KLF13 | -0.80 | 6.98E-06 | 8.37E-04 |
| CCR2 | -0.77 | 2.53E-05 | 2.00E-03 |
| SASH3 | -0.70 | 1.17E-07 | 5.74E-05 |
| SOS1 | -0.64 | 4.33E-07 | 1.36E-04 |
| CD8B | -0.64 | 1.98E-06 | 3.66E-04 |
| CRTAM | -0.64 | 3.50E-07 | 1.15E-04 |
| TGFBR1 | -0.62 | 9.22E-05 | 4.67E-03 |
| TOB2 | -0.61 | 3.10E-07 | 1.07E-04 |
| FYN | -0.61 | 3.49E-05 | 2.53E-03 |
| CRKL | -0.60 | 4.50E-07 | 1.40E-04 |
| CD244 | -0.60 | 1.82E-04 | 7.30E-03 |
| ITGB2 | -0.60 | 1.95E-03 | 3.49E-02 |
| IKZF3 | -0.57 | 6.86E-05 | 3.86E-03 |
| SEC24C | -0.56 | 1.10E-07 | 5.44E-05 |
| MYO1F | -0.54 | 3.92E-05 | 2.71E-03 |
| SPN | -0.54 | 9.48E-05 | 4.73E-03 |
| NFATC2 | -0.54 | 5.64E-05 | 3.42E-03 |
| SIT1 | -0.54 | 5.22E-09 | 5.86E-06 |
| IL16 | -0.53 | 6.31E-06 | 7.83E-04 |
| TUBB | -0.51 | 4.97E-07 | 1.45E-04 |
| TNFSF14 | -0.50 | 4.97E-07 | 1.45E-04 |
| LCP2 | -0.50 | 2.63E-07 | 1.01E-04 |
| XRCC4 | 0.16 | 2.16E-04 | 8.17E-03 |
| PRKCA | 0.23 | 2.77E-06 | 4.67E-04 |
| TAX1BP1 | 0.27 | 9.39E-05 | 4.70E-03 |
| CDC42 | 0.29 | 9.24E-05 | 4.67E-03 |
| PSMA1 | 0.30 | 4.99E-06 | 6.83E-04 |
| PIAS1 | 0.31 | 2.27E-04 | 8.46E-03 |
| PIK3CA | 0.35 | 7.33E-04 | 1.82E-02 |
| SAMHD1 | 0.37 | 3.58E-09 | 4.77E-06 |
| CD83 | 0.42 | 2.71E-05 | 2.11E-03 |
| TCF12 | 0.43 | 1.19E-06 | 2.57E-04 |
| PURB | 0.44 | 4.44E-05 | 2.94E-03 |
| DUSP4 | 0.45 | 7.34E-04 | 1.82E-02 |
| SP3 | 0.49 | 6.05E-06 | 7.64E-04 |
| CXCR4 | 0.50 | 2.68E-04 | 9.35E-03 |
| SP100 | 0.52 | 4.81E-07 | 1.42E-04 |
| HMGB1 | 0.53 | 1.88E-05 | 1.69E-03 |
| SOD2 | 0.56 | 7.69E-05 | 4.15E-03 |
| BCL10 | 0.56 | 8.34E-05 | 4.38E-03 |
| AIMP1 | 0.70 | 7.41E-06 | 8.63E-04 |
| TIPARP | 0.74 | 5.12E-06 | 6.87E-04 |
| HMGB2 | 0.74 | 1.05E-08 | 9.77E-06 |
| PELI1 | 0.83 | 8.90E-07 | 2.14E-04 |
| TNFAIP3 | 1.13 | 5.47E-05 | 3.36E-03 |
| RGS1 | 1.17 | 9.56E-05 | 4.75E-03 |
| IFI44L | 1.19 | 5.12E-05 | 3.22E-03 |


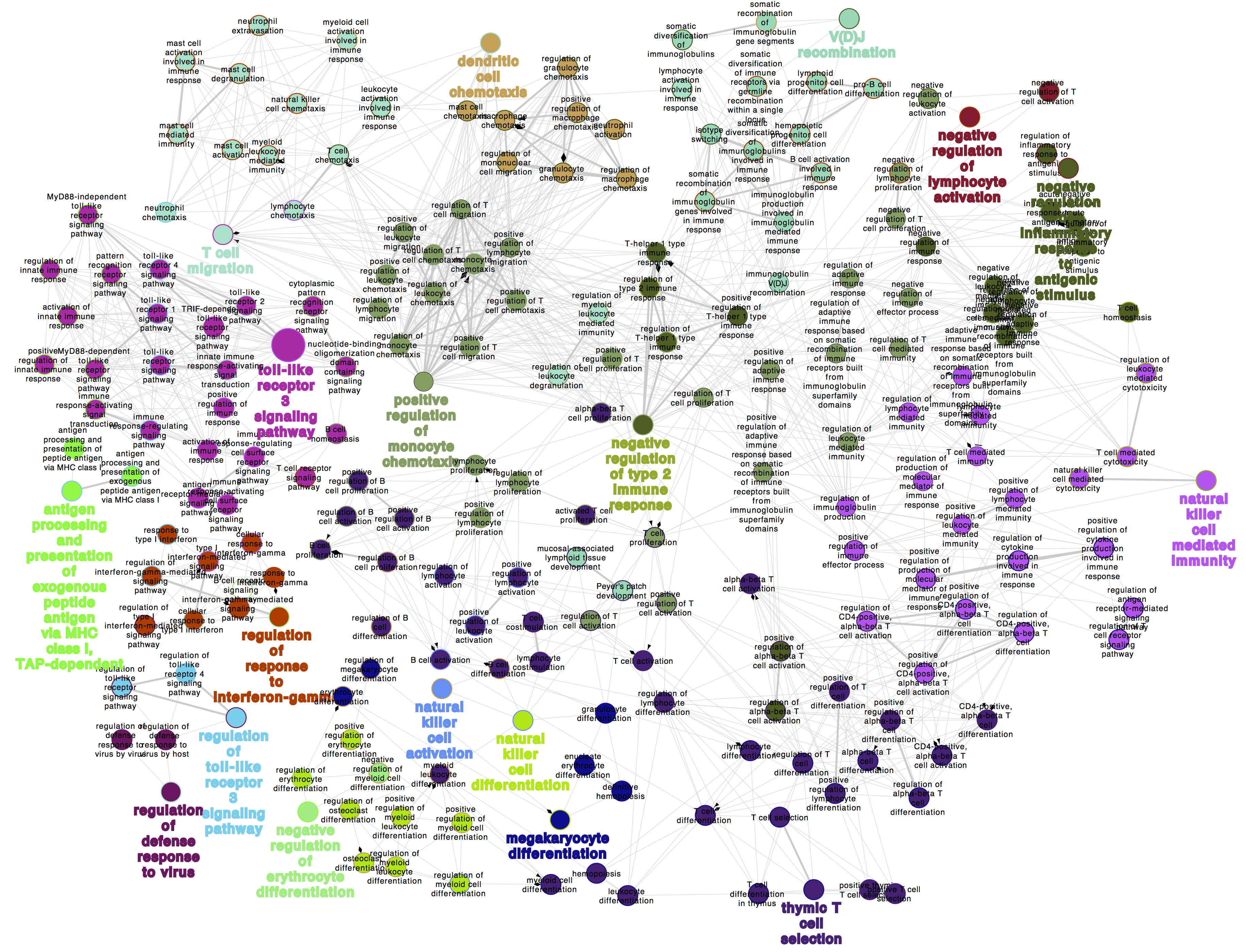


**Supplemental Figure 1.** The identified target genes of the differentially expressed microRNA were cross referenced against the differentially expressed mRNAs between MS patients and controls resulting in 920 overlapping genes. These genes were classified according to function by GO and 100 of these genes were classified as being involved in immune processes. They were further analyzed using functional module enrichment based on Immune System Gene Ontology which resulted in 21 modules.
